# The Implementation of Federated Digital Identifiers in Health Care: Rapid Review

**DOI:** 10.2196/45751

**Published:** 2024-02-08

**Authors:** Karishini Ramamoorthi, Vess Stamenova, Rebecca H Liu, Onil Bhattacharyya

**Affiliations:** 1 Institute for Health System Solutions and Virtual Care Women's College Hospital Toronto, ON Canada; 2 Department of Family and Community Medicine University of Toronto Toronto, ON Canada

**Keywords:** digital identity, electronic health record, environmental scan, identity management, identity verification, national electronic health record, online access, PAEHR, patient records, patient-accessible electronic health records

## Abstract

**Background:**

Federated digital identifiers (FDIs) have been cited to improve the interoperability of data and information management while enhancing the privacy of individuals verifying their identity on the web. Many countries around the world have implemented FDIs in various sectors, such as banking and government. Similarly, FDIs could improve the experience for those wanting to access their health care information; however, they have only been introduced in a few jurisdictions around the world, and their impact remains unclear.

**Objective:**

The main objective of this environmental scan was to describe how FDIs have been established and implemented to enable patients’ access to health care.

**Methods:**

We conducted this study in 2 stages, with the primary stage being a rapid review, which was supplemented by a targeted gray literature search. Specifically, the rapid review was conducted through a database search of MEDLINE and Embase, which generated a list of countries and their services that use FDIs in health care. This list was then used to conduct a targeted gray literature search using the Google search engine.

**Results:**

A total of 93 references from the database and targeted Google searches were included in this rapid review. FDIs were implemented in health care in 11 countries (Australia, Belgium, Canada, Denmark, Estonia, Finland, Iceland, Norway, Singapore, Sweden, and Taiwan) and exclusively used with a patient-accessible electronic health record system through a single sign-on interface. The most common FDIs were implemented nationally or provincially, and establishing them usually required individuals to visit a bank or government office in person. In contrast, some countries, such as Australia, allow individuals to verify their identities entirely on the web. We found that despite the potential of FDIs for use in health care to facilitate the amalgamation of health information from different data sources into one platform, the adoption of most health care services that use FDIs remained below 30%. The exception to this was Australia, which had an adoption rate of 90%, which could be correlated with the fact that it leveraged an opt-out consent model.

**Conclusions:**

This rapid review highlights key features of FDIs across regions and elements associated with higher adoption of the patient-accessible electronic health record systems that use them, like opt-out registration. Although FDIs have been reported to facilitate the collation of data from multiple sources through a single sign-on interface, there is little information on their impact on care or patient experience. If FDIs are used to their fullest potential and implemented across sectors, adoption rates within health care may also improve.

## Introduction

### Overview

Medical information is increasingly available to users through the digitization of health care [1]. This is reshaping how patients interact with the health care system by facilitating information exchange with providers and institutions. Today, patients who receive care from different organizations access their personal health information through multiple patient portals, and this information is stored separately without one place to organize or process the information [2]. Contributing to this fragmented system is the use of decentralized identities, which require patients to create, verify, and remember multiple usernames and passwords for the services owned and controlled by multiple distinct entities [3]. Although having unique electronic credentials may be perceived by some as being more secure, users are prone to creating the same logins and passwords for multiple applications in a “do-it-yourself” password management strategy, which negates the perceived increase in security [4]. This ultimately impacts the quality of the user experience. To mitigate this issue, federated identity management solutions are being introduced into health care from other sectors to improve the interoperability of health care data among institutions [3]. These solutions use federated digital identifiers (FDIs) as part of the identity proofing process, which involves binding a user with their credentials (such as a driver’s license) and using that information for the authentication process [4]. Authentication is performed by a trusted identity provider, who then uses the identity proof to verify that the user is who they claim to be. Once the user has been authenticated, a relying party authorizes the user to access their services [5]. FDIs can reduce repetitive logins and the need to remember multiple passwords for patients, which reduces the number of siloed systems and facilitates a continuum of care that enables improved health care decision-making [4]. In fact, according to the Digital Identity and Authentication Council of Canada, interoperable health management systems that maintain patient privacy and autonomy can be achieved by using FDIs [6]. A good FDI permits authentication of a person’s identity and has the following features: (1) It needs to be verified and authenticated with high assurance (ie, the initial registration process is accomplished with high standards), (2) it needs to be unique such that each individual only has 1 identity within a system, (3) it must be established with an individual’s consent so that users are aware of what personal data is being shared, and (4) it must protect the user’s privacy and allow them to control how they use their personal data [6,7]. Some researchers have even said governments have a formal responsibility to ensure that the digital identity infrastructure will not result in the disempowerment of individual citizens [8]. Considering the boom in the digital identity market, developing trustworthy processes and infrastructure is key in order to avoid security risks that can occur or are created at a rapid speed and on a large scale [9].

Many governments have implemented FDIs in various sectors, including health care. However, only a few countries (like Estonia and Australia) have applied their citizen FDI across multiple sectors (eg, financial, government, and health care) [6,7]. In contrast, other countries have applied it narrowly, and its use within health care tends to be lower [8]. Barriers to widespread adoption across sectors have been said to include user trust, unintended effects that may arise from requirements needed to establish an FDI, and policies and regulations [1]. As a result, FDIs have largely matured in the financial and government sectors, while their use in health care remains in its infancy [6]. Therefore, as the use of patient-facing digital platforms increases and more jurisdictions aspire to develop patient-centric systems, there is a need to understand how FDIs have been used, specifically in health care. Previous reviews largely focus on the implementation of platforms that use FDIs to provide patients with access to health care services, and they place an emphasis on factors such as policies, stakeholder engagement, and infrastructure [7]. Alternatively, the reviews that aim to understand how FDIs are used in health care focus on the login procedures used by patients to access their own data [8-11]. Therefore, there is a lack of studies that aim to understand how the FDIs used in health care are established or how their use impacts the implementation and uptake of the platforms that leverage them [7,12]. Therefore, given that governments globally have recently placed an emphasis on designing and implementing FDIs that transform how citizens access their data, we sought to characterize how FDIs being used in health care are currently established and leveraged to integrate different data sources [1,4,6,12].

### Objectives

This rapid review aims to explore how FDIs have been established and implemented globally to provide patients with access to health care resources and services.

## Methods

This environmental scan was conducted in 2 stages: a literature review using a rapid review methodology, followed by a targeted gray literature search [13].

### Literature Review

#### Search Strategy

We conducted a literature search in the MEDLINE and Embase databases on February 1, 2021, to identify services that leveraged digital IDs within a health care setting. Our search strategy was built using key terms surrounding the concepts of “national electronic health records,” “digital identity,” and “single sign-on” (Multimedia Appendix 1 contains the detailed search strategy). Upon identifying services that applied digital identifiers to access health data, we also performed targeted searches on MEDLINE and Embase using the names of these services. All articles were exported into Zotero (Corporation for Digital Scholarship, Roy Rosenzweig Center for History and New Media), a citation management software.

#### Article Screening

For the literature search, as per commonly used rapid review methodologies [13-15], a 2-step screening consisting of a title-abstract and full text screening was performed by a single reviewer (KR). During the first step of screening, articles were excluded based on the title, and abstracts were read in cases where the title was not conclusive. During the full-text screening stage, all articles were read in detail. The inclusion of papers was restricted to full-text publications in the English language published between January 1, 2011, and February 1, 2021, since articles examining FDIs in health care were uncommon before 2011. Specifically, since 2011, there has been a general upward trend in the total number of related articles. Articles focusing on the implementation and deployment of FDIs or single sign-on services within health care were included, along with those that focused on stakeholder experiences when interacting with these services. Research articles, commentaries, reviews, and nonresearch articles were included. Articles that focused on the implementation of a national electronic health record without a patient access component or articles that did not describe how the FDI was used in health care were excluded, as our focus was on the patients’ user experience and the implementation of the FDI for health care purposes.

A standard data extraction form was used by a single reviewer (KR) to extract the following details from the included studies: author, year, country, name and features of the patient-accessible electronic health record (PAEHR), consent model, infrastructure, name and type of digital identity, process citizens use to access the data, name and type of identity provider, year implemented, and adoption rate. A 10% quality check was completed by a second reviewer (VS), as per common rapid review methodologies [13], wherein 10% of articles selected randomly were screened by VS based on the title, then abstracts, and when needed, full texts were consulted if additional details were required. Any discrepancies in the inclusion and exclusion of articles were discussed. Although this rapid review does not entail a quality assessment, a narrative approach consistent with the data extraction of a rapid review was used [13]. All data extractions were also discussed between KR and VS.

#### Gray Literature Search

The literature review generated a list of countries and services that have implemented FDIs to access health care resources. We used this list to conduct an additional gray literature search using Google (Multimedia Appendix 1). The Google search results were limited to the first 10 pages, allowing us to focus our data collection on the most relevant identified services from their respective service and government websites. For the supplemental gray literature search, a single reviewer (KR) screened the search results and extracted the data.

### Ethical Considerations

This review has not been registered, and a protocol has not been developed.

## Results

### Literature and Gray Literature Search

**Figure 1:**
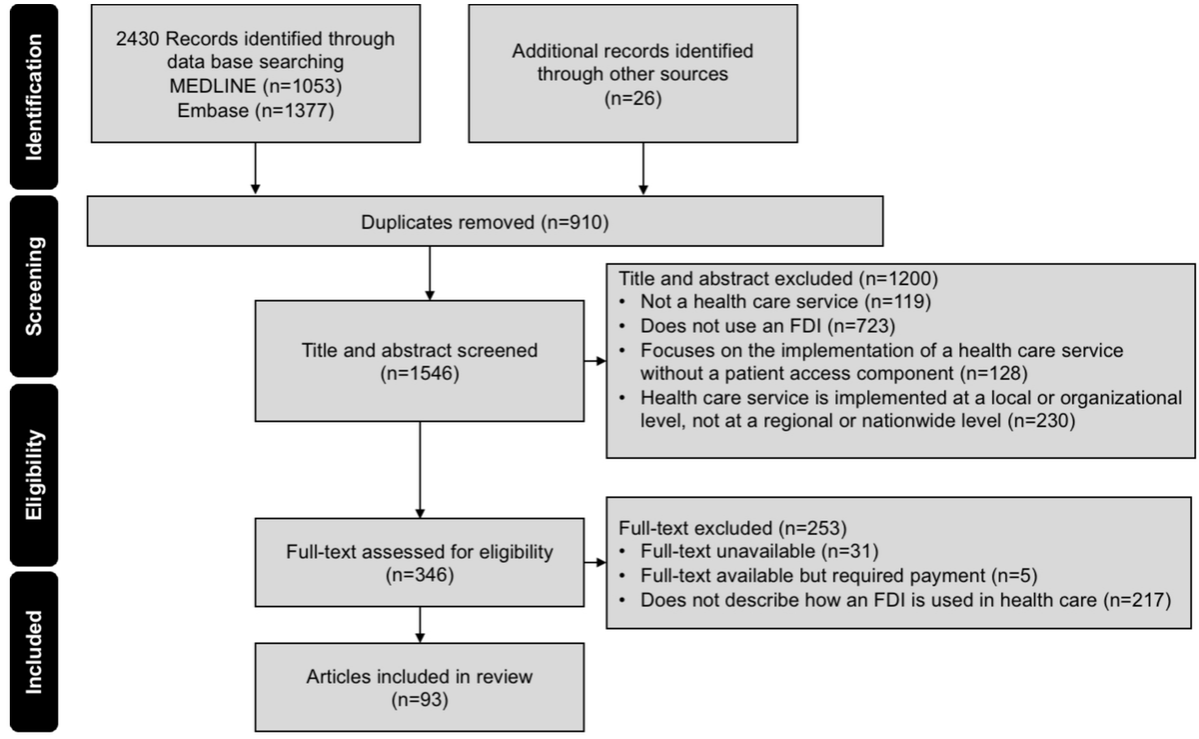
PRISMA (Preferred Reporting Items for Systematic Reviews and Meta-Analyses) diagram detailing the search and selection process applied to the rapid review. FDI: federated digital identifier.

The results of the search are summarized in a PRISMA (Preferred Reporting Items for Systematic Reviews and Meta-Analyses) flow diagram in Figure 1. A total of 93 references were included in the review [10,11,16-106].

Through our literature search, we identified 9 countries that used digital identifiers for citizens’ access to health care information. Our targeted searches identified 2 additional countries, for a total of 11 countries identified using FDIs. These were Estonia, Finland, Australia, Taiwan, Denmark, Norway, Sweden, Iceland, Singapore, Canada, and Belgium.

### Application of FDIs to Health Care Platforms

Among the 11 identified countries, the most common application of digital identifiers in health care was in the context of the national PAEHR. The exception to this was Canada, where citizens of Alberta and Quebec access their health care information on a provincial level because health care is provincially managed [17,76]. Most countries that have implemented digital identifiers at a national level are in Europe.

Identifiers can be verifiable (ie, they can be used to authenticate a citizen’s identity) and reusable (ie, they can be used across multiple sectors to provide citizens with access to multiple services) [9,16]. Across the jurisdictions we identified, the FDIs were always verifiable, and all except for Taiwan were also reusable [10,17,74]. Countries used 1 of 2 implementation frameworks [9]. Most countries (eg, Australia) use a centralized implementation framework that facilitates the collation of data from existing repositories and data sources into a central storage system [9]. Distributed implementation frameworks were used less frequently (eg, in Denmark, Sweden, and Iceland), and they stored the data presented on the single sign-on interface in disparate repositories and pulled relevant data into the system only when required [9].

Table 1 provides a comparison of how patients receive access to their health care services through a national or provincial PAEHR that uses FDIs. The earliest use of digital IDs for patient access was in Denmark in 2003 [9,11].

**Table 1:** Overview of countries around the world that have implemented a federated digital identifier to enable patient access, the name of the federated digital identifier, and the consent model used by the country.

Country; name of PAEHR^a^	Year of implementation	Description of platform	Consent model	FDI^b^ used for access	References
Estonia; Digilugu.ee	2009	Centralized her system, which contains information about every interaction a patient has with the health care system. This is visible to both patients and to all clinicians who treat them.	Opt-out	Estonian eID	[10,11,16-21]
Finland; My Kanta Pages	2010	A web-based service where citizens can view data entered by private and public health care services concerning their interactions with the health care system as well as data they have entered themselves.	Opt-out except for ePrescription	Banking ID, mobile ID, or chip-based identity card enabling web-based transactions	[10,11,22-29]
Australia; My Health Record	2012	A web-based summary of citizens’ key health information in one place. Health care providers can also see the record when they need to (eg, in an emergency).	Opt-out	MyGovID	[10,11,30-72]
Taiwan; My Health Bank	2014	A web-based service that allows people with National Health Insurance to view their own medical history.	Opt-in	National Health Insurance Card (NHI Card)	[17,73,74]
Denmark; Sundhed.dk	2003	A public portal that gives citizens and health care providers access to and information about all Danish health care services.	Opt-out	NemID	[9,11,26,75-78]
Norway; Helsenorge	2011	A web-based portal that provides citizens access to health care services and digital access to documents from patient portals.	Opt-out	BankID, BuyPass ID, or Commfides e-ID	[10,11,75,79,80]
Sweden; Journalen	2012	A web-based national patient portal that allows patients access to all of their health care information and is compatible with the electronic health record used by any of the patient’s providers.	Opt-in	Freja eID plus or Bank ID	[10,11,26,77,81,94]
Iceland; Heilsuvera	2014	A portal that provides educational material about health, including prevention, while allowing citizens to manage their health care information.	Not Reported	Electronic ID (eID)	[11,95]
Singapore; HealthHub	2015	A one-stop health service that provides access to a family’s health records, health information, and services.	Not Reported	SingPass ID	[96-99]
Canada; Carnet Santé Quebec	2018	A web-based service that provides access to citizens’ health information.	Opt-in	clicSÉQUR	[26,100,101]
Canada; MyHealth Records Alberta	2019	A single account that provides citizens access to store health information in a secure place.	Opt-in	MyAlberta Digital ID	[11,102-104]
Belgium; Summarised Electronic Health Record	2019	A set of documents that health professionals, in consultation with the patient, decide to share as they are deemed necessary and relevant for care.	Not Reported	Bankcard, or Belgian ID	[105,106]

^a^PAEHR: patient-accessible electronic health record.

^b^FDI: federated digital identifier.

### Establishing and Verifying FDIs

IDs were usually obtained at a municipal office, a government service center, a post office, or a bank that served as the identity provider. Among the jurisdictions studied, only Australia and Taiwan allowed individuals to obtain and verify a digital identity entirely on the web [45,74]. In contrast, the other jurisdictions (Belgium, Denmark, Estonia, Iceland, Norway, Singapore, Sweden, and Quebec) required citizens to obtain further documentation in person or had information mailed to their home address as an additional step to web-based verification [19,24,45,76,79,86,101,102,107].

Among the jurisdictions included in the study, Table 2 summarizes the common forms of identification used by identity providers in establishing a digital ID. The most common documents used to verify one’s identity when establishing a digital ID were a passport or banking information. Other common methods included jurisdictional ID cards and a driver’s license.

**Table 2:** Forms of verification used by various identity providers when establishing a digital identifier.

Infrastructure and country; name of PAEHR^a^ [references]		FDI^b^ used for access	Login ID verification
**Centralized infrastructure^c^**			
	Estonia; Digilugu.ee [10,11,19-21]	Estonian eID	Government ID and mobile ID
	Finland; My Kanta Pages [10,11,24]	Banking ID, mobile ID, or chip-based identity card enabling web-based transactions	Bank ID and mobile ID
	Australia; My Health Record [10,11,45-47]	MyGovID	Government ID and additional verification using health care or banking information
	Taiwan; My Health Bank [73,74]	NHI Card (Not reusable)	Other
**Distributed infrastructure^d^**			
	Denmark; Sundhed.dk [10,11,72]	NemID	Government ID
	Norway; Helsenorge [10,11,79,80]	BankID, BuyPass ID, or Commfides e-ID	Government ID, bank ID, mobile ID, and other
	Sweden; Journalen [11,17,86]	Freja eID plus or Bank ID	Government ID, bank ID, and mobile ID
	Iceland; Heilsuvera [95]	eID	Government ID and mobile ID
**Infrastructure not reported**			
	Singapore; HealthHub [99]	SingPass ID	Government ID
	Canada; Carnet Santé Quebec [101]	clicSÉQUR	Government ID and additional verification using Health Insurance number to obtain an activation code
	Canada; MyHealth Records Alberta [11,102]	MyAlberta Digital ID	Government ID and additional verification using Alberta Health Card number
	Belgium; Summarised Electronic Health Record [106]	Bankcard, or Belgian ID	Government ID, bank ID, and additional verification using the identification token and verification code sent to mobile phone

^a^PAEHR: patient-accessible electronic health record.

^b^FDI: federated digital identifier.

^c^Centralized PAEHR uses a central store and is implemented as a separate layer on top of electronic health records that are already in use.

^d^Distributed PAEHR encourages health information exchange between different electronic health records and other data sources without creating a central store.

Different countries also instated either an opt-in or opt-out consent model for their PAEHRs. Opt-in consent models required users to agree to participate in the program, whereas in an opt-out consent model, users were automatically enrolled in the service and had to “opt-out” from the service to be excluded [11,42]. Some countries that used an opt-out consent model used it strategically to improve the adoption of their PAEHR platform [50]. For example, Australia switching from an opt-in to an opt-out consent model correlated with an increase in adoption from 20% to 90% [11,50].

### Integrated Health Services in Platforms Using FDIs

Table 3 compares the features found in various PAEHR platforms that use FDIs through a single sign-on interface to provide patients access to their health information. Most platforms provide citizens with the ability to share and restrict access to their records or portions of their records to specific providers and provide a log of all personnel that have accessed their records. In some locations, such as Alberta and Estonia, health care providers can only access a specific user’s information if they already have an existing patient-provider relationship [10,104]. In contrast, citizens in Australia cannot restrict access to the summary portion of their health record but can limit what is included in the summary, who can see the additional documents outside the summary, and who can remove documents if they want to [10,51]. In addition, most platforms, such as the Journalen in Sweden, allow citizens to share their health information with other users (eg, family members) [82]. They also often provide the ability for patients to view their record, or in some instances, such as in Australia’s My Health Record, to view and edit their record to provide information on their personal health summary or to provide advance care directives [10,11,35,56]. Other features commonly available to citizens on most platforms include the ability to access their laboratory records, medications, and clinical summaries [11].

**Table 3:** Features of patient accessible electronic health record platforms that use federated digital identifiers (n=12). The most commonly implemented features include access to laboratory records, medications, and clinical summaries.

Features [references]		PAEHRs with feature, n (%)
**Access to health information**		
	Access logs [10,11,17,23,75,96,100,103,105]	10 (83)
	Share or restrict access to providers [10,11,17,23,73,105]	9 (75)
	Provide access to trusted individual [10,11,23,96,103]	8 (67)
	Parental access to child’s record [10,11,17,23,96]	7 (58)
**Appointment information**		
	Visits (time, date, or provider) [10,11,17,23,96,103]	9 (75)
	Book appointments [10,11,17,103]	8 (67)
	Referrals [23,75]	6 (50)
**Reports or notes**		
	Clinical summaries [10,11,17,96,103,105]	10 (83)
	Laboratory results [10,11,17,23,74,96,100,105]	10 (83)
	Medical reports (radiology, pathology, etc) [17,23,75,96,105]	9 (75)
	Diagnoses and conditions [10,11,17,96,103,105]	8 (67)
	Child growth and development [17,51]	5 (42)
**Medication information**		
	Medication and dispensing [10,11,17,96,100,103,105]	11 (92)
	Allergies and adverse reactions [23,51,96,103,105]	7 (58)

### Adoption Rates

**Figure 2:**
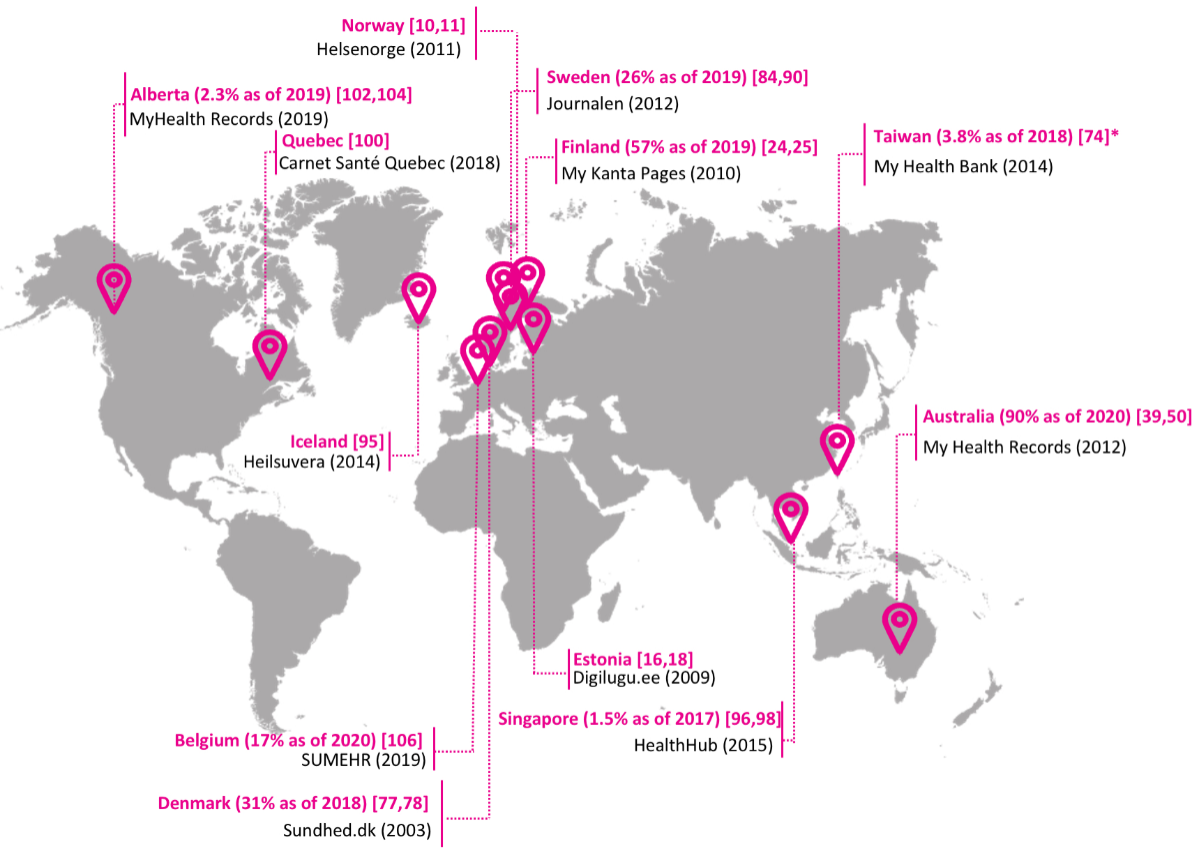
Countries and provinces (adoption rate in parentheses where available) and their respective PAEHR (year implemented in parentheses) that use federated digital identifies to provide patients with access to their health records and the names of their respective patient-accessible electronic health records. The adoption rate for Taiwan (920,000/23,726,185) was calculated as a percentage of the total population [74].

The adoption rates of these PAEHR services that used FDIs varied considerably around the world (Figure 2; [10,11,16,18,24,25,39,50,74,77,78,84,90,95,96,98,100,102,104,106]). Australia and Singapore had the highest and lowest adoption rates at 90% and 1.5%, respectively, and most countries had adoption rates below 30% [23,40].

## Discussion

### Overview

This study found 11 countries that leveraged FDIs with a health care platform to provide patients access to their health information. FDIs were typically established and implemented by the government on a national level, and they were always used in the context of a PAEHR. PAEHRs allow patients to access data from multiple health care services through a single sign-on interface using either a centralized implementation framework (most common) or a distributed framework (less common). Most authentication procedures to verify a person’s identity when establishing an FDI (to use with the single sign-on interface) were completed in person, and the identity providers most often included the bank or government. Additionally, with the exception of Taiwan, all countries used a reusable FDI, meaning the digital identity could be used in other sectors as well. Overall, the studies in the literature do not focus on describing patients’ experiences with the FDI login and registration process but rather on the experience with the platforms these FDIs provided access to, that is, the PAEHRs.

### Comparison to Other Work

To our knowledge, this is the first environmental scan of FDIs that describes how they are established and used in health care to provide patients with access to their health information. Previous reviews have only described user experiences focusing on their interaction with the PAEHR platforms themselves, as opposed to the experience of creating an FDI and using it to access their personal health information [10,11,108]. Although understanding these user experiences can inform factors that impact the uptake of the platform itself, it does not speak to the factors that impact the uptake of the digital ID or the influence that the digital ID creation experience has on the overall experience of the PAEHR user. Therefore, given that FDIs are increasingly being explored as an option to improve patient access to health care information [1,8], it is important for health system leaders to understand what identity verification and authentication processes are commonly used globally today. This will, in turn, inform how leaders design FDI establishment processes in the future.

### Factors to Consider When Implementing FDIs in Health Care

One of the main perceived benefits of FDIs is their ability to reduce repetitive logins, eliminate the need to remember multiple passwords, and facilitate care across different institutions [6]. Given that FDIs used in the context of a national PAEHR do not require separate logins to access their health care information to begin with since electronic access to health care is already consolidated into 1 platform (from the users’ perspective), the unique benefits of FDIs may not be as apparent. In these situations, the benefit of implementing an FDI may derive from its use across sectors (eg, financial and other governments), preventing users from having to remember different login information for their different needs (eg, banking vs health care logins). Therefore, in these situations, the true benefits of FDIs only become apparent if one assesses their benefits across sectors [109,110]. In countries where the health care system is more fragmented and where there are multiple institutional, reimbursement, and regional portals, the benefits of FDIs to the health care sector alone may become more obvious [109,110]. Specifically, implementing FDIs across different jurisdictions could drive adoption of digital identity since it increases the value of having a digital identifier. This could, in turn, drive users to use the services that leverage the digital ID to provide access. Our environmental scan found that current literature is limited to describing how FDIs are established (ie, identity verification process using banking information, a passport, etc) or how they are used to provide access to health care services (ie, the login procedures) [11,17] However, there were no studies that aim to understand users’ perceptions of the process of establishing an FDI and users’ experiences with using an FDI when accessing health care resources. This makes it difficult to understand the barriers and facilitators to the adoption of FDIs in health care settings.

### Factors Affecting PAEHR Adoption Irrespective of FDI Use

Through this environmental scan, we hoped to ascertain how patients perceive the benefits of FDIs, but as most FDIs have been used in the context of PAEHRs, the literature focused on how patients perceive the use of their country-specific PAEHR as opposed to the use of the FDI that enabled access. Common PAEHR platform features included providing patients with access to their laboratory and medical reports, booking appointments, and allowing a trusted individual to access their health care information [10,17,24]. The adoption rates of these PAEHR platforms remained below 30% in most countries even years after implementation, and this was true irrespective of the type of identity management being used (federated or not) [110,111]. Similar trends are also observed in countries such as France and the United Kingdom, which have also implemented PAEHRs for the entire nation but provide access without an FDI [111,112]. The adoption rates in these countries have been as low as 0.5% and 1.5%, respectively [111,112]. Our environmental scan found that among the countries that used FDI-managed PAEHRs, platforms with an opt-out consent model, such as Australia and Finland, generally had higher adoption rates [51,113]. This strategy to improve PAEHR adoption has also been reported in non-FDI PAEHR platforms [110]. Similarly, the factors that influenced adoption of PAEHRs were not unique to platforms that use FDIs with a single sign-on interface and were instead common to all digital health technologies. For example, facilitators such as supportive legislation, clear government guidelines, recognized standards, and proper stakeholder engagement worked across PAEHR platforms [114-117]. Negative attitudes and beliefs about health care professionals, a lack of leadership engagement, a lack of comprehensive information, stakeholder disagreements, and the presence of multiple local initiatives were, on the other hand, common barriers across PAEHRs, irrespective of the type of identity management system used [108,117,118].

Our environmental scan found that the information available to patients on the PAEHR platforms using FDIs varied in terms of who had access to the information, what information was presented, and how the information could be changed and modified [23,35,54,82]. For example, an individual in Taiwan was able to share or restrict information access to specific health care providers but could not provide access to a trusted individual like a caregiver, whereas someone from Singapore was unable to restrict access to a specific provider that belongs to their care circle but was able to provide access to an individual that they trust [18,74,97,98]. This variation can also be observed in PAEHRs not managed by FDIs [111,117]. Previous work by Essen et al [10] examining policy documentation of PAEHRs around the world also highlighted a heterogenous PAEHRs landscape with distinct services, regulation approaches, and patients’ access across jurisdictions.

### Limitations

All instances of FDI use in the literature were in the context of PAEHRs, with no studies focused exclusively on FDIs in the context of health care, so it was difficult to identify what benefits related to the use of FDI versus. those related to the use of PAEHR in general. We have reported the features and adoption rates of PAEHRs in this study, as it may be that the use of FDIs makes most sense in the context of regional PAEHRs. The low rate of adoption of these platforms could be attributed to challenges with the implementation and adoption of PAEHRs, not necessarily with the use of FDIs. Among the 11 countries that we identified as using FDIs for health care access, 46 of 93 included studies describing Australia’s My Health Record, which was the most developed platform with the highest adoption rates among those reviewed. In comparison, the PAEHRs in other countries were not well established or studied, which made broader generalizations more limited. Finally, as this was an environmental scan with a rapid review, we only reviewed 2 databases and only included resources available in English, so it is possible that some platforms that leverage FDIs for health care access were missed. To mitigate this issue, we supplemented our academic search with gray literature and included governmental reports and websites in our resources.

### Conclusion

Federated digital IDs have been leveraged for health care use around the world as their use facilitates the amalgamation of information through a single sign-on interface, which allows patients to access their information from multiple data sources. FDIs have been used exclusively in the context of PAEHRs, and adoption of PAEHRs remains low in many countries. As a result, it is difficult to disentangle the unique contribution of FDIs to these adoption rates. As FDIs provide patients with the opportunity to have a single point of access to health care services and information from multiple sources, future studies could focus on exploring patients’ perceptions about the benefits and drawbacks of FDIs specifically. If FDIs are used to their fullest potential and implemented across sectors, adoption rates within health care may improve.

## Appendix

Multimedia Appendix 1 Search strategy.

## Abbreviations

FDI: federated digital identifier
PAEHR: patient-accessible electronic health record
PRISMA: Preferred Reporting Items for Systematic Reviews and Meta-Analyses

## Data Availability

The data sets generated during and analyzed during this study are available from the corresponding author on reasonable request.
