# Supplementary material for: The Implementation of Federated Digital Identifiers in Health Care: Rapid Review
Source: J Med Internet Res. 2024 Feb 8;26:e45751. doi: 10.2196/45751 (PMC10884916; doi:10.2196/45751)
Supplement: Multimedia Appendix 1 [file jmir_v26i1e45751_app1.doc]

*The Implementation of Federated Digital Identifiers (FDIs) in Healthcare: An Environmental Scan*

*SUPPLEMENTAL*

*Search Strategy*

| 1. digital identity.tw,kf or identity management.tw,kf or identity verification.tw,kf or blockchain/ or (blockchain).tw,kf or . single sign-on.tw,kf or central authentication system.tw,kf or security assertion markup language.tw,kf or integrated health information system.tw,kf or citizen access.tw,kf |
| --- |
| 2. national electronic health record.tw,kf. or national EHR.tw,kf. or national electronic medical record.tw,kf. or national EMR.tw,kf. or national health informat* system*.tw,kf. or national medic* informat* system.tw,kf. |
| 3. limit (1 or 2) to (english language and yr="2011 -Current") |

*Search strategy used for supplemental grey literature search:*

[Location] AND (“digital ID”) AND [Name of PAEHR]

Systematically substituted with the following terms:

| *Location* | *Name of PAEHR* |
| --- | --- |
| Australia | MyHR |
| Belgium | SumEHR |
| Denmark | Sundhed.dk |
| Estonia | Digilugu.ee |
| Finland | My Kanta Pages |
| Iceland | Heilsuvera |
| Norway | Helsenorge |
| Singapore | HealthHub |
| Sweden | Journalen |
| Taiwan | My Health Bank |
| Canada (Alberta) | MyHealthRecords |
| Canada  (Quebec) | clicSEQUR |
